# Neuromuscular Responses to 5 K Time Trial Load Carried by Spanish Army Marines

**DOI:** 10.3390/sports13040129

**Published:** 2025-04-21

**Authors:** Beltrán Cáceres-Diego, Pedro E. Alcaraz, Cristian Marín-Pagán

**Affiliations:** 1 UCAM Research Center for High Performance Sport, UCAM Universidad Católica de Murcia, 30107 Murcia, Spain; palcaraz@ucam.edu (P.E.A.); cmarin@ucam.edu (C.M.-P.); 2 Strength and Conditioning Society, 30008 Murcia, Spain; 3 Facultad de Deporte, UCAM Universidad Católica de Murcia, 30107 Murcia, Spain

**Keywords:** military, soldier, military personnel, load carriage, neuromuscular fatigue, performance, physical readiness and conditioning

## Abstract

One of the physical requirements for marines involves covering a specific distance while carrying individual combat gear, supplies, or other military equipment across challenging terrain. Training for this physically and mentally demanding task is a routine component of their preparation. However, further research is needed to better understand the neuromuscular impact of such demanding efforts, strenuous maneuvers, and the recovery process in the subsequent days. Twenty-nine marines completed a 5 km time trial while carrying 24 kg of combat gear, undergoing evaluations at four time points: pre exercise, immediately post exercise, 24 h post exercise, and 48 h post exercise. Repeated measures ANOVA, paired samples *t*-test, and effect size (ES) analysis were conducted, presenting the results as the mean ± standard deviation (SD). The significance level was set at *p* ≤ 0.05. Several variables and their corresponding *p*-values demonstrated changes over time, including the following: the isometric mid-thigh pull (IMTP) (*p* = 0.001); countermovement jump height (VJ CMJ) (*p* ≤ 0.001); rating of fatigue scale (ROF) (*p* ≤ 0.001); blood lactate (BL) levels (*p* ≤ 0.001); maximum pull-ups (PUmax) (*p* ≤ 0.001); body mass (BM) (*p* ≤ 0.001); dominant hand grip strength (DHGS) (*p* = 0.406); and non-dominant hand grip strength (NDHGS) (*p* = 0.805). Incident reports and perceived effort (IRPE) revealed a progressive and significant increase between the first and last kilometer of the test, specifically in perceived variables of fatigue, muscle pain, joint pain, shortness of breath, palpitations, excessive sweating, and muscle tremors (all *p* ≤ 0.001). These findings may contribute to optimizing training programs to better align with operational demands, thereby improving task performance and overall mission effectiveness. In conclusion, the military test had a significant neuromuscular impact on the body, initially resulting in the potentiation of absolute global isometric strength and lower-limb power. However, these values declined below the baseline levels at 24 and 48 h post test.

## 1. Introduction

The primary duties of any military force include the defense of the nation, protecting its citizens, and maintaining peace, both nationally and internationally. Within the military, specialized units carry out a wide range of responsibilities, each playing a crucial role in achieving these overarching objectives. Among the most operationally active military units is the infantry, whose members must be prepared to face and overcome any adversity, no matter how dangerous, by adapting to hostile and unpredictable environments. To successfully fulfill their mission, infantry soldiers require a high level of physical and mental conditioning to meet the demands of their operational duties.

Essentially, infantry soldiers are required to move from one point to another across terrain that is inaccessible to vehicles, all while carrying their combat gear. Over time, this equipment has increased in both size and weight due to the incorporation of technologically advanced combat and supply items intended to enhance protection against evolving threats [1]. As a result, the minimum physical and operational readiness standards required to perform such duties have also risen. In some cases, mission success depends on how rapidly soldiers can reach a designated location, or on their ability to survive by reaching the extraction point in the shortest possible time while carrying their gear. This operational necessity has given rise to one of the most physically demanding military tasks: the loaded march. Loaded marches are an essential component of military training maneuvers, requiring a high level of physical exertion [2]. This task, which involves covering a specific distance while carrying combat gear and equipment in the shortest possible time, demands significant neuromuscular and aerobic fitness, with an emphasis on maximum strength and absolute maximal oxygen consumption (VO_2_max) [3]. Due to the high physical demands, soldiers often experience a notable decline in their physical performance, which can persist for several days after the activity [4]. This decline may manifest as reduced muscle strength, accumulated fatigue, and prolonged recovery, highlighting the importance of proper recovery strategies and physical readiness in military contexts.

This test is routinely conducted by the Marine Infantry as part of their military training, as it is a mandatory component of the Spanish Marine Infantry’s training and instruction program. The objective of the test is to ensure that marines can cover a specified distance with all the necessary equipment and instruments for mission success in under thirty minutes. The test simulates an offensive action where a soldier must perform combat maneuvers while carrying individual equipment over a set distance. It also replicates scenarios in which an operational team must reach an extraction point in a safe zone within the designated timeframe.

The conditions under which these tests are conducted in a military environment, similarly to those of a real mission, are seldom ideal. Previous studies have shown that stress factors affecting operational performance in military personnel [5,6] include both intrinsic and extrinsic variables. Intrinsic factors involve body composition, gender, age, prior training experience, initial physical performance level, injury history, lifestyle, and psychosocial variables, while extrinsic factors include sleep deprivation, negative energy balance, the work/rest ratio, social and environmental influences, and the methods and materials used in prolonged physical activity training. These extrinsic factors significantly influence the occurrence of overuse injuries in military personnel due to their inability to withstand or mitigate the stress of training loads [7]. Moreover, running while carrying a load is influenced by specific performance limitations that contribute to energy expenditure and, consequently, fatigue during exercise. These limitations include the mass of the load, the individual’s body mass, load distribution, movement speed, plate carrier vest, terrain type, and weather conditions [8,9].

These tests are often conducted with accumulated fatigue from previous days and may be followed by additional military activities as part of training exercises, typically with minimal rest between activities, and limited sleep and food intake. These conditions, common in military environments, can affect performance during the test and influence the loaded march’s impact on the body, potentially impairing the efficiency of subsequent activities in the following days. This can also increase the risk of injury, as the body may not fully recover from the initial exertion. It is well known that military tasks, activities, and training [10], particularly among infantry soldiers, increase the likelihood of overuse injuries due to three primary scenarios [11]: (a) a rapid increase in training load following a period of inactivity or low activity; (b) taking on a training load that exceeds an individual’s capacity; and/or (c) prolonged participation in high-level training programs, leading to musculoskeletal tissue degradation.

Despite the valuable insights from previous studies [12,13], a significant gap remains in the scientific literature regarding experimental research. While literature reviews are abundant, experimental studies are scarce, with most failing to adequately simulate the complex environmental factors and operational demands faced by military personnel during training and deployment. This study offers an innovative perspective thanks to it being carried out under real operational conditions, diverging from the artificial settings commonly used in prior research. Unlike earlier studies conducted in controlled, laboratory-based environments, this investigation was carried out during an official Spanish Army test that replicates the conditions of an actual mission or deployment. The test was designed specifically to assess the physical and operational performance of military personnel, involving a distance greater than the commonly used two-mile benchmark. Furthermore, the participants were experienced marines, fully trained and in optimal physical and mental condition on the day of the test. The equipment and weaponry used were identical to those employed in real deployment scenarios, thereby ensuring the high ecological validity of the results. These elements collectively provide the unique value of the present study within the scientific literature.

Few studies have documented how military-specific tests (10–20 km, 18–61 kg) impact the neuromuscular and cardiorespiratory systems, as well as performance in specific military tasks [14,15,16,17]. There is a lack of comprehensive research analyzing the combined effects of carried load, running speed, and real-world conditions, as these variables are often overly controlled or oversimplified in laboratory settings. It has been demonstrated that VO_2_max, maximal strength, and muscular endurance are highly correlated with running while carrying low to moderate loads of 15–25 kg [18], with the general assumption that this correlation is proportional to the load carried, speed, and duration of the test. Research has shown that carried load and movement speed contribute most significantly to fatigue during loaded running [9], with the data indicating that fatigue is delayed during long-duration loaded marches when performed at intensities below ~47% of VO_2_max or ~4.6 km/h [8]. Running speed, in particular, plays a crucial role in influencing fatigue and performance. The speed at which personnel move during loaded marches or runs significantly impacts neuromuscular fatigue, energy expenditure, and overall performance [8,19]. Given that military personnel frequently operate at varying speeds and distances in unpredictable environments, understanding how speed interacts with load and fatigue in real-world scenarios is crucial for optimizing training protocols and enhancing operational readiness.

Therefore, given the need for more experimental research that replicates real-world conditions to better understand the effects of load and speed on performance, recovery, and overall resilience in military contexts, further studies are required to determine how these physically and psychologically demanding tests impact the neuromuscular system and the recovery process in the days following their completion. Understanding the physical demands of loaded running tests, their effects on the body, and the recovery from fatigue in the days following their completion through neuromuscular performance tests conducted before and after the trial could help to optimize training load management. This could ensure peak performance during these tests and facilitate the consolidation of physiological adaptations. Therefore, this study aimed to analyze and describe the acute and residual neuromuscular fatigue resulting from a 5 km time trial while carrying 24 kg of combat gear.

## 2. Materials and Methods

### 2.1. Study Design

A descriptive repeated-measures study was conducted with 29 professional military personnel, involving a 5 km time trial on flat terrain using a 500 m circuit and 24 kg of combat load. Evaluations were conducted prior to the test as a baseline reference, immediately after the test to assess the acute effects on short-term fatigue and neuromuscular performance, and again at 24 and 48 h post test to observe the impact, potential residual fatigue, and recovery trajectory in relation to physical performance and operational readiness. The total distance covered, elevation gain, and other variables of the 5 km time trial were recorded using identical smart sports watches equipped with GPS technology, providing detailed data. Before the trial, the supervising and command team measured the total course distance to ensure its accuracy. The trial characteristics are expressed as mean ± SD. The total weight of the combat load was 24 kg, consisting of a 24 h survival backpack, helmet, rifle, boots, and a plate carrier with magazine pouches. Participants underwent a familiarization session with the tests prior to the study’s implementation. Participants refrained from engaging in vigorous exercise during the 24 h preceding the study. Following the completion of a general warm-up and a subsequent specific warm-up, pre-training data were collected, including participants’ age, height, and body mass (BM). The assessment was an official exercise administered by the military academy as part of its scheduled maneuvers. The planning and workload were meticulously tailored and specifically adapted for execution on the day of the test, ensuring that the soldiers were fully prepared and mentally focused to perform at their best. As an official and evaluative test integral to the academy’s program, it was not an external supplementary evaluation. In addition, the following performance tests were conducted in the predetermined order: blood lactate (BL), countermovement jump (CMJ), isometric mid-thigh pull (IMTP), hand grip strength (HGS), and rating of fatigue scale (ROF). A specific test was also included: maximum pull-ups in 2 min (PUmax). These tests were conducted in the specified order, with 3–5 min of rest between each. Immediately after completing the time trial, the data were re-collected, and the performance tests were repeated (post test) to assess acute fatigue. Measurements were also taken 24 and 48 h post test to evaluate residual fatigue. Additionally, an incident report and perceived effort (IRPE) was compiled at the end of the test.

### 2.2. Participants

The sample consisted of 29 male participants (age: 28.7 ± 4.7 years; height: 176.2 ± 5.4 cm; body mass: 78.4 ± 8.0 kg), all of whom were sergeant trainees at the Spanish Marine Infantry School. The inclusion criteria were as follows: (a) active-duty military personnel, (b) belonging to the CAES (access course to the non-commissioned officer scale) of the Marine Infantry Corps, and (c) the absence of any diagnosed illness, injury, or deficiency. The exclusion criteria included (a) the presence of any diagnosed illness, injury, or deficiency that could impair physical performance during the study, (b) undergoing pharmacological treatment potentially affecting physical capacity, (c) failure to complete all phases and protocols of the study, and (d) voluntary withdrawal from the study at any point. The sample consisted exclusively of male participants, as there were no women in the CAES during the study period. Prior to the start of the study, all participants were informed about the nature and procedures of the intervention and provided written informed consent documents in accordance with the Helsinki Declaration [20]. The study was approved by the Ethics Committee of the Catholic University of San Antonio of Murcia (UCAM), as well as by the commanding officers of the Marine Infantry School “General Albacete y Fuster” (Ethics approval code: (CE102202, 28 October 2022).

### 2.3. Procedures

Warm-up: Prior to each data collection session, participants completed a standardized warm-up protocol comprising the following elements: (a) joint mobility exercises: 1 set of 10 repetitions for each of the following movements: shoulder rotations, trunk rotations, hip twists, and standing knee flexion forward; (b) dynamic stretching: 1 set of 10 repetitions for each of the following exercises: standing hip flexion extension with ground contact, standing hamstring stretch lifting the leg to horizontal and touching toes with the hand; (c) metabolic activation exercises: 1 set of 8–20 repetitions for each of the following exercises: jumping jacks, skipping, lunges, and burpees; and (d) specific strength exercise: barbell deadlifts: set 1 (4 × 20 kg), set 2 (6 × 40 kg), and set 3 (4 × 60 kg), with 1–2 min of rest between sets.

Maximum isometric leg and back strength: The isometric mid-thigh pull (IMTP) is a well-established and reliable method for assessing an individual’s overall maximal strength [21]. Participants performed the IMTP using a Smith machine placed on a portable force platform (Kistler 9286BA, Kistler Group, Winterthur, Switzerland). The barbell was fixed in position, and its height was adjusted to be equidistant between the greater trochanter and the lateral epicondyle of both knees. Prior to data collection, the device was calibrated according to the manufacturer’s specifications to ensure accuracy and validity. Participants first completed a preliminary submaximal trial (~70% effort) lasting 3–5 s. Following this, they performed two maximal effort attempts, with 20 s of rest between each repetition. Verbal encouragement was provided to ensure maximum effort. The highest recorded value was used for subsequent analysis.

Countermovement jump (CMJ): The countermovement jump (CMJ) is one of the most extensively validated, reliable, and widely used tests for assessing lower-limb power and strength [22]. The CMJ was performed on a portable force platform equipped with four piezoelectric sensors located at each corner, capable of conducting stabilometric analysis via center of pressure oscillations. The platform had a measurement range of 400–600 mm, and the sampling frequency was set 1000 Hz, allowing for the precise recording of displacement and foot contact time with the platform surface. Prior to measurements, the device was calibrated according to the manufacturer’s instructions to ensure accurate and valid measurements. The data were recorded and analyzed using Kistler’s measurement, analysis, and reporting software (MARS, 2012 S2P Ltd., Ljubljana, Slovenia), which provides stable and reliable squat-derived parameters. MARS has proven effective for individual monitoring and for detecting performance changes over time [23]. Participants were allowed to self-select the depth of the countermovement and were instructed to land as close as possible to their take-off point. Each participant performed two jumps, separated by a one-minute rest interval. The best jump, determined by the maximum vertical height achieved at take-off, was used for analysis. The raw data were exported, and the following parameters were calculated using Microsoft Excel (Microsoft Corporation, Redmond, WA, USA): maximum vertical jump height (VJ) in centimeters [cm], peak power (PP) in watts [W], peak eccentric (PEcc) and concentric (PCon) force in newtons [N], and rate of force development (RFD) for both the eccentric (RFDEcc) and concentric (RFDCon) phases, measures in newtons per second [N/s].

Rating of fatigue scale (ROF): Participants were asked to rate their perceived level of fatigue after completing the test using a numerical scale from 0 to 10, where 0 represents a state of complete rest and 10 denotes maximum effort or exhaustion [24]. This score was used to quantify the subjective perception of fatigue experienced by each participant in response to the physical exertion of the trial.

Blood lactate (BL): BL levels were assessed only in the post-test phase. For pre-test comparison, standardized resting lactate values were used as a reference [25]. Post-test BL was measured using a capillary blood sample obtained from the index finger of the right hand. The site was first cleaned of sweat and then disinfected with alcohol-wetted gauze. A puncture was performed using a sterile, single-use lancet. The first drop of blood was discarded to avoid contamination, and the second drop was collected and applied to a test strip, which was immediately inserted into a portable lactate analyzer (model Lactate Pro 2, Arkray, Kyoto, Japan) for measurement.

Maximum pull-ups in 2 min (PUmax): This test, widely used for decades to assess muscular strength and/or endurance in military personnel [6,26], required participants to perform the maximum number of pull-ups within a two-minute time limit, with the chin rising above a horizontal bar. Pull-ups were executed with a pronated grip, with the hands positioned at or wider than shoulder width. The initial position involved hanging from a horizontal bar with elbows fully extended and feet off the ground. Each repetition had to be completed by pulling the body upward until the chin was above the bar, specifically ensuring that the Adam’s apple passed the top of the bar [27,28]. The test concluded upon either reaching volitional exhaustion or completing the two-minute duration. Participants were required to maintain continuous grip on the bar throughout the test, without descending or making contact with any surface for rest. Participants were informed that repetitions performed with incorrect or poor technique would not count. A brief rest period of less than 10 s was permitted while maintaining grip. The maximum number of valid repetitions was recorded for analysis.

Hand grip strength (HGS): HGS is a fundamental test used to assess performance in manual lifting and carrying tasks, which are common in military contexts [29]. It was measured using a digital hand dynamometer (Camry model EH101, Zhongshan Camry Electronic Co., Ltd., Zhongshan, China) to determine the maximum isometric grip strength. This variable has been shown to correlate strongly with general health indicators, clinical outcomes, and total body strength [30]. During testing, the shoulder of the evaluated arm remained close to the torso, with the elbow flexed at 90° and the wrist in a neutral position. Two maximal-effort attempts were performed for each hand, dominant (DHGS) and non-dominant (NDHGS), with each contraction held for 3 s. A rest interval of 30 to 60 s was allowed between attempts. The highest value recorded for each hand was used for statistical analysis.

Incident reports and perceived effort during test (IRPE): Upon completion of the 5 km time trial, participants completed a report documenting any incidents, discomfort, symptoms, or subjective perceptions experienced during the test. This included self-reported ratings of fatigue, muscle pain, joint pain, shortness of breath, palpitations, excessive sweating, and muscle tremors. Ratings were given using a four-level ordinal scale: 0 (none), 30 (slight), 60 (moderate), 90 (severe), and were recorded separately for each 1 km segment of the test (0–1 km, 1–2 km, 2–3 km, 3–4 km, and 4–5 km). Additionally, an open-ended observation section was also provided, allowing participants to describe any notable incidents or difficulties encountered during the 5 km test.

### 2.4. Statistical Analyses

Statistical analysis was conducted using Jamovi software, version 1.6 (The Jamovi project, Sydney, Australia). Inferences were calculated based on the magnitude of fluctuations in performance variables at pre test, post test, 24 h post test, and 48 h post test. The results were expressed as standardized mean differences (mean ± standard deviation, SD) [31]. The normality of the data was evaluated, confirming that all variables met the established criteria. The Shapiro–Wilk test was used for this assessment, considering both the *p*-value and the W statistic. To evaluate the effects of time, group, and their interaction, a repeated-measures analysis of variance (ANOVA) was performed, including multiple comparisons between different time points and training protocols (time × training protocols). Subsequently, each studied variable was compared across different time points using paired samples *t*-tests. The significance level was set at *p* ≤ 0.05. The effect size (ES) was calculated using Cohen’s d correlation coefficient [32] by comparing pre-test to post-test and pre-test to 24 h post-test values. This measure was used to determine the significance of the effect of each variable, reflecting meaningful differences across different time points. The effect size (ES) was interpreted as Cohen’s d correlation coefficient with the following classifications: trivial (<0.2), small (≥0.2–< 0.6), moderate (≥0.6–<1.2), large (≥1.2–<2.0), very large (≥2.0–<4.0), and extremely large (≥4.0) [33]. In summary, the guidelines for the use of statistics in sports medicine and exercise sciences were adhered to [33].

## 3. Results

Regarding the march characteristics collected using similar smart sports watches from 12 participants, the data are presented as mean ± SD. The total distance of the test was initially measured by the supervising and command team to ensure accuracy. The total distance covered was 4.97 ± 0.05 km. The total time was 28.06 ± 2.87 min. The average running pace was 5.65 ± 0.61 min per kilometer. The total estimated calories burned were 416.25 ± 77.67 kcal, calculated based on the individual’s anthropometric characteristics, physical condition, and the activity performed, such as age, height, body mass, gender, type of activity, duration, intensity, and heart rate during the test. The average heart rate was 171.08 ± 16.69 beats per minute, with peak rates reaching 187.67 ± 14.54 bpm. The average temperature during the trial was 27.66 ± 1.12 °C, with maximum temperatures reaching 29.7 ± 1.0 °C. The average speed was 10.75 ± 1.13 km/h, with maximum speeds reaching 13.59 ± 1.35 km/h. The total time for the 29 participants who completed the trial, expressed as mean ± SD, was 28.86 ± 3.35 min.

**Figure A1:**
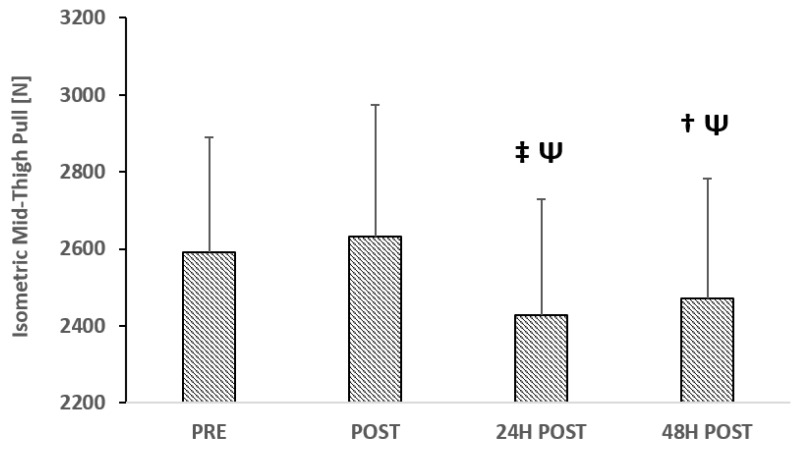
Evolution of results for the isometric mid-thigh pull test. Values are represented as means ± SD. The following symbols indicate significant differences compared to values: (a) pre: † = significant difference (*p* ≤ 0.01), ‡ = very significant difference (*p* ≤ 0.001) and (b) post: Ψ = very significant difference (*p* ≤ 0.001).

The IMTP test revealed significant changes over time (*p* < 0.001) in the absolute peak vertical force (see Figure A1, Appendix A.1). This force increased by 1.67% immediately post test, but showed a significant decrease of −6.29% at 24 h post test, and −4.57% at 48 h post test compared to the pre-test values.

**Figure A2:**
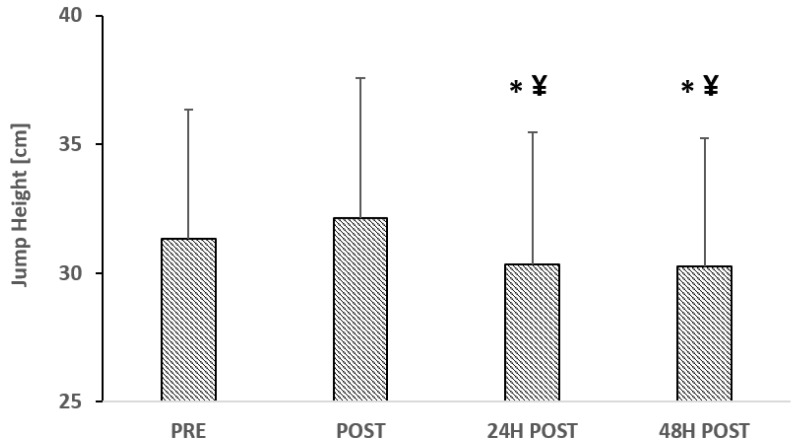
Evolution of results for the countermovement jump height test. Values are represented as means ± SD. The following symbols indicate significant differences compared to the values: (a) pre: * = significant difference (*p* ≤ 0.05) and (b) post: ¥ = significant difference (*p* ≤ 0.01).

Regarding the CMJ variables, the VJ showed significant decreases across different time points (*p* < 0.001) (see Figure A2, Appendix A.2), as did the PEcc (*p* < 0.001) and RFDEcc (*p* < 0.001). However, no notable changes were observed in the PP (*p* < 0.082), PCon (*p* = 0.844), or RFDCon (*p* = 0.132). The evolution of changes in the CMJ variables varied across different time points. Specifically, the VJ showed no significant change from pre test to post test (2.46%), but decreased by −3.25% at 24 h post test and −3.51% at 48 h post test compared to the baseline values. The PEcc exhibited a notable decrease of −7.68% immediately post test, with a slight recovery of 0.21% at 24 h and an increase of 3.51% at 48 h compared to the pre-test values. The RFDEcc decreased by −14.93% from pre test to post test, followed by a significant increase of 8.46% at 24 h and 19.58% at 48 h compared to the pre-test values. In contrast, the following variables showed no significant changes: the PP decreased by −3.52% from pre test to post test, −7.58% from pre test to 24 h post test, and −5.12% from pre test to 48 h post test. The PCon decreased by −0.51% from pre test to post test, −0.78% from pre test to 24 h post test, and 0.24% from pre test to 48 h post test. Finally, the RFDCon increased by 9.21% immediately post test, but decreased by −18.74% at 24 h and −20.62% at 48 h compared to the pre-test values.

**Figure A3:**
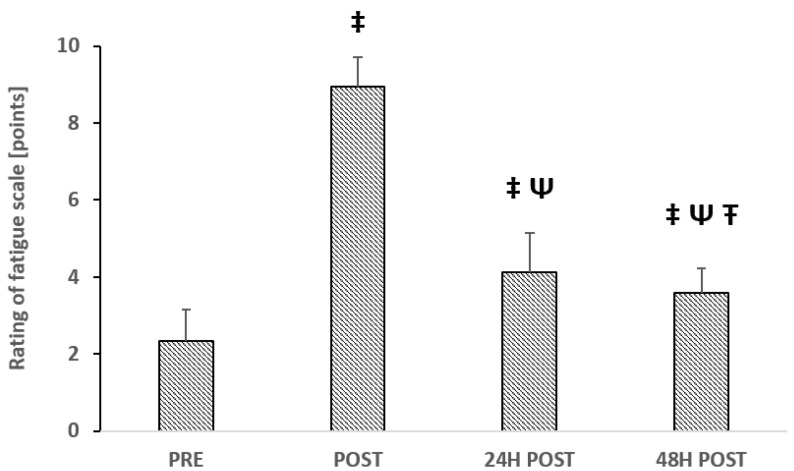
Evolution of results for the rating of fatigue scale test. Values are represented as means ± SD. The following symbols indicate significant differences compared to the values: (a) pre: ‡ = significant difference (*p* ≤ 0.001), (b) post: Ψ = significant difference (*p* ≤ 0.001) and (c) Post 24 h: Ŧ = significant difference (*p* ≤ 0.01).

**Figure A4:**
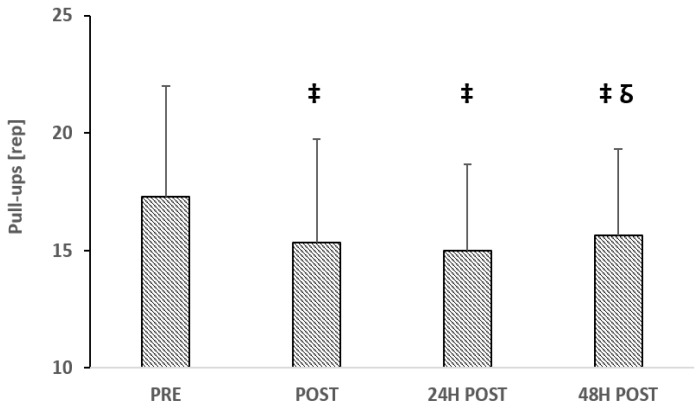
Evolution of results for the pull-up test. Values are represented as means ± SD. The following symbols indicate significant differences compared to the values: (a) pre: ‡ = significant difference (*p* ≤ 0.001) and (b) post 24 h: Ᵹ = significant difference (*p* ≤ 0.05).

The performance variable results are summarized in Table 1. The ROF showed a significant increase over time (*p* < 0.001), with a 282.48% increase immediately post test (see Figure A3, Appendix A.3). This was followed by a gradual decrease of 76.07% at 24 h and 53.42% at 48 h compared to the pre-test values. The BL test demonstrated a significant increase (*p* < 0.001) from pre test to post test, with an increase of 378.64%. The BM showed significant changes over time (*p* < 0.001), remaining below the baseline values by −0.55% immediately post test and −0.59% at 24 h post test. However, it returned to baseline levels (+0.01%) at 48 h post test. The PUmax showed significant decreases across different time points (*p* < 0.001) (see Figure A4, Appendix A.4), with reductions of −11.23% from pre test to post test, −13.19% from pre test to 24 h post test, and a slight recovery of −9.38% at 48 h post test, though still below baseline values.

**Figure A5:**
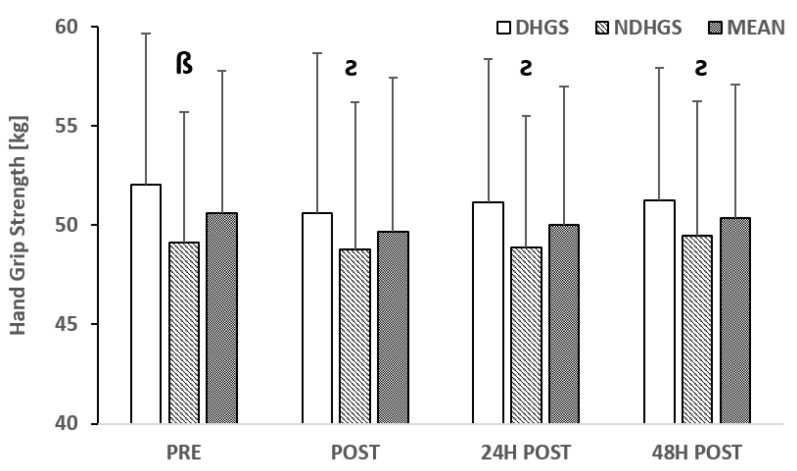
Evolution of results for the hand grip strength test. Values are represented as means ± SD. The following symbols indicate significant differences between DHGS (dominant hand grip strength) and NDHGS (non-dominant hand grip strength): ƨ = significant difference (*p* ≤ 0.05), and ß = significant difference (*p* ≤ 0.001).

The HGS did not show significant overall changes over time (*p* = 0.647) (see Figure A5, Appendix A.5). When analyzing each hand individually, the DHGS also did not exhibit significant changes over time (*p* = 0.406), though it decreased by −2.77% immediately post test, −1.67% at 24 h, and −1.50% at 48 h compared to the baseline values. Similarly, the NDHGS did not vary significantly over time (*p* = 0.805), with decreases of −0.73% pre–post, −0.53% pre–post 24 h, and a slight increase of +0.69% pre–post 48 h. In contrast, there were significant differences between the dominant and non-dominant hands (*p* = 0.004), with the mean DHGS being 51.29 ± 7.30 and the mean NDHGS being 49.05 ± 6.78, resulting in a −4.57% difference. The difference in grip strength between the two hands was significant at various time points: −5.94% (*p* = 0.001) pre test, −3.77% (*p* = 0.038) post test, −4.73% (*p* = 0.020) 24 h post test, and −3.70% (*p* = 0.043) 48 h post test.

Data from the IRPE assessment are compiled in Table 2. In general, the number and severity of symptoms and incidents increased with the distance covered. Significant changes (*p* < 0.001) were observed across different segments of the test for all evaluated parameters, including perceived fatigue, muscle pain, joint pain, shortness of breath, palpitations, excessive sweating, and muscle tremors.

The incidents recorded in the post-test report that impaired test performance were, broadly speaking, attributed to fatigue and insufficient recovery prior to the test, as well as several cases of muscle strain and pain in the soles of the feet, chest, quadriceps, and lower back. Additionally, more than 70% of participants reported clear signs of fatigue and performance deficits due to high temperatures on the day of the test, along with symptoms of dehydration, excessive sweating, dizziness, nausea, severe headaches, and reduced running caused by heat exposure.

## 4. Discussion

To gain new insights into neuromuscular fatigue and recovery after a 5 km time trial while carrying 24 kg of combat gear under operational military conditions, this study aimed to analyze and describe the neuromuscular impact of the trial and the progression of fatigue over the following days. The primary finding was that the test initially caused an increase in overall absolute isometric strength and lower-body power immediately after completion. However, this was followed by significant neuromuscular fatigue, with no evidence of full recovery after 48 h.

Despite the high physical demands of the test, the post-IMTP values increased by nearly 2% compared with the baseline ones, before remaining significantly reduced at both 24 and 48 h. A previous study [17] observed a significant decrease in lower-body strength after a 20 km march with loads of 34, 48, and 61 kg. In contrast, the increased post-test values observed in the present study may be attributed to the heightened psychophysiological activation of the sympathetic nervous system, likely caused by the stress of the test [34]. This reversible physiological and psychological response to stress leads to altered cortical excitation, which in turn increases heart rate, muscular strength, energy metabolism, cortisol levels, and body temperature, while also distorting the perception of fatigue [35,36]. Such overexcitement could be critical for survival in actual combat scenarios, where success or failure may mean the difference between life and death. However, caution should be exercised before engaging in further submaximal efforts, as the neuromuscular system is likely to experience considerable fatigue following such activities. Accumulated fatigue can impair neuromuscular control during dynamic movements, potentially increasing the strain on other structures and raising the risk of musculoskeletal injury due to overuse [37,38]. Additionally, unconscious variations in motor performance may occur as a protective mechanism to prevent overloading tissues involved in repetitive or continuous tasks [39], which could further contribute to increase the risk of injury.

Similarly, the VJ measured during the CMJ initially increased following the test, but then significantly decreased at 24 and 48 h. Given that the test likely induced less metabolic fatigue compared to other long-duration exercises, it is possible that the high mechanical tension resulting from repeated eccentric actions to absorb BM, combined with the additional load carried during each stride and the significant involvement of the eccentric phase in high-speed running, contributed to post-activation potentiation, which counteracted the induced fatigue [40]. A similar effect was observed in study [41] on sub-elite long-distance runners, which found that after performing a vigorous training session consisting of 4 × (3 × 400 m/1′)/3′, more than half of the participants experienced post-activation potentiation in strength and power. This suggests that trained athletes are able to maintain their work capacity despite significant exertion, as evidenced by the results from both the IMTP and VJ tests. Performance in this discipline appears to be influenced not only by metabolic adaptations, but also by specific neuromuscular adaptations resulting from the type of training. Similar findings have been documented previously [42], where VJ increased, while peak torque strength decreased following a maximal running test. Another study observed that after completing a 10 km march while carrying loads of 18, 27, and 36 kg, VJ and maximum power increased by 13.05%, 8.51%, and 2.36%, respectively [14]. The results obtained in the present study, when compared to other long-duration tests with heavier loads, highlight the role of average running speed, which translates to a higher exercise intensity. Military training is typically characterized by high volumes of exercise at low intensity; therefore, incorporating higher-intensity sessions is necessary to target these metabolic pathways while also enhancing muscle fiber recruitment, in accordance with the size principle. This adaptation enables greater muscle strength and power, even after prolonged exertion, potentially improving performance in real-world missions by enhancing central nervous system arousal.

Other variables assessed in the CMJ test, such as the PP, exhibited a gradual decline following test completion, with a slight recovery observed at 48 h. Similarly, the PEcc and RFDEcc both decreased immediately after the test, but values at 24 and 48 h indicated supercompensation above the baseline levels. In contrast, the RFDCon increased immediately after the test, but then declined at 24 and 48 h compared with the baseline values, while the PCon remained reduced at all time points, showing only a slight increase at 48 h post test. Previous research has demonstrated that during running, the eccentric actions of the support foot contribute to enhancing concentric propulsive actions by absorbing mechanical energy and converting it into elastic energy via stretch-shortening cycles, thereby improving running economy [43,44]. Eccentric actions contribute more significantly than concentric actions to mechanical performance in terms of force and power, thus enhancing propulsion. This difference becomes more pronounced as movement speed increases [45]. In this study, the average speed during the test was 10.8 km/h, with peak speeds reaching 13.6 km/h. It has been demonstrated that at 4.6 km/h (~45% VO_2_max), there is an inflection point at which energy expenditure increases substantially [8]. Therefore, it is plausible that the demands of the test, characterized by numerous eccentric actions, whose intensity was amplified by both movement speed and the additional carried load [46], led to the post-test decline in the PEcc and RFDEcc, followed by supercompensation and an enhanced stretch-shortening cycle. This is evidenced by the increased RFDCon and supercompensated PCon values after the test [47].

The ROF values showed a substantial increase in subjective fatigue after the test, rising by approximately 282.5%. However, recovery was relatively rapid, with values returning closer to baseline at 24 and 48 h. The number and severity of symptoms and incidents escalated as the distance covered increased, with notable symptoms including fatigue, shortness of breath, and excessive sweating. Correspondingly, the BL levels rose by nearly 378.6% compared with the pre-test values. Although the test was primarily aerobic in nature [2], the elevated lactate levels suggest a significant reliance on carbohydrate metabolism [48], as well as a reduced ability to clear accumulated lactate due to exercise intensity exceeding the maximum lactate steady state. To sustain performance and mitigate the risk of dehydration, implementing a refreshment station providing food and hydration during the test would be highly beneficial, particularly in high-temperature conditions, similar to those observed in this study.

Furthermore, environmental factors exacerbated the difficulty of the test. Elevated temperatures contributed to premature fatigue in all participants, negatively affecting their performance. This was reflected in the BM measurements, which exhibited a significant decrease lasting up to 24 h before returning to baseline values at 48 h. The observed reduction in BM suggests that participants did not experience severe dehydration. However, improved recovery strategies may be necessary, as BM remained below baseline 24 h post test. Additionally, the inclusion of a refreshment station with food and water would have been an effective strategy to counteract the adverse effects of elevated temperatures, particularly on exceptionally hot days.

The progression of the PUmax across different evaluations highlights the negative impact of the 5 km loaded run with 24 kg on performance in specific military tasks, as all mean values at various time points remained below the baseline levels. In line with these findings, previous studies [14,15,17] have reported a significant decline in shooting accuracy and grenade-throwing distance following marches of 10–20 km with loads ranging from 34 to 61 kg. Given these results, it is crucial for individuals undergoing similar tests to prioritize effective recovery strategies to restore performance levels as quickly as possible. This is particularly important when engaging in repeated exertion over consecutive days, such as during training maneuvers or extended military exercises.

Grip strength values for both hands (DHGS and NDHGS) remained below the baseline levels across all time points, with no significant changes observed. However, a slight increase above baseline was noted at 48 h for the non-dominant hand. Previous research has similarly reported reduced isometric grip strength for up to 72 h following eight days of military maneuvers [49]. As in the aforementioned study, this reduction in grip strength may be attributed to diminished neural drive, as well as decreases in firing rate, motor unit synchronization, or recruitment [50], likely due to the fatigue induced by the test. Additionally, a disparity of approximately 5% was observed between the dominant and non-dominant hands. While this deficit does not reach the threshold associated with increased injury risk (10–15%) [51], bilateral deficits can still pose a limitation during physical exertion and elevate the likelihood of injury, particularly when performing efforts near maximal capacity. Strength training has been shown to mitigate these imbalances, thereby enhancing physical performance in military-specific tasks and reducing injury risk [52].

The primary limitation of this study was the inability to standardize food and fluid intake before, during, and after the test, which would have ensured uniformity in dietary intake across participants. Additionally, the sample size may have been limited due to the physical demands of the test. For individuals required to perform load-bearing running tests, it is essential to possess a strong foundation of general muscular strength to support the specific demands of load-carrying running activities. This foundational strength is crucial for absorbing the high ground reaction forces generated by the repeated impacts of running, which increase at higher velocities. Therefore, it is imperative to collaborate with a strength and conditioning coach who can design a tailored training program to optimize test performance, ensure effective recovery, and support progressive training development.

Potential future research directions building upon the present study could include the evaluation of similar military tests or tasks under real-world conditions, incorporating recovery periods extending beyond 48 h post assessment, with a focus on monitoring performance and neuromuscular recovery. Additionally, future studies could investigate the impact of such tests on the endocrine system when performed under stress conditions that closely replicate those encountered in operational environments. Furthermore, research could aim to quantify or propose nutrient intake strategies before, during, and in the days following the test, with the objective of enhancing performance in the test itself or in specific military tasks. Alternative approaches to accelerating neuromuscular recovery after the test could also be explored, with the goal of restoring optimal combat readiness in the shortest possible time.

Finally, regarding practical relevance and the implications for military training and real-world operational environments, our findings may contribute to the development of training programs that incorporate longer recovery periods, particularly following the completion of highly demanding physical tests under adverse environmental conditions, given the incomplete recovery observed at 48 h. Furthermore, the implementation of neuromuscular performance monitoring would significantly aid in identifying individuals who tolerate and recover adequately from training load-induced stress, as well as those who do not, thereby helping to reduce the risk of musculoskeletal injuries. This approach would also be particularly appropriate prior to engaging in other physically demanding military activities or maneuvers. Moreover, our findings suggest that military tasks, and the sequence in which they are performed, should be strategically prioritized, as the substantial fatigue induced by such tests can negatively impact the execution and effectiveness of subsequent military operations.

## 5. Conclusions

In conclusion, the military test imposed significant physical and mental demands, particularly under adverse weather conditions involving high temperatures. It had a considerable neuromuscular impact on the body, initially resulting in the potentiation of global isometric strength and lower-body power immediately after the test, followed by a decline in most of the analyzed variables and inadequate neuromuscular recovery 48 h post test. Therefore, it is essential to implement a periodized physical training program tailored to the specific needs of military personnel engaged in high-demand physical activities, such as those evidenced in this study. This program should be planned in advance, with appropriate training load management and sufficient rest, to ensure that service members are in optimal condition and fully prepared to perform at their best when required. The observed potentiation following such a physically demanding test may have resulted from the increased activation of the neuroendocrine system due to the stress associated with combat-like scenarios, such as defending a position or reaching an extraction point in hostile terrain. The tests conducted in this study were designed to evaluate performance in critical aspects of military duties, including endurance, muscular strength, and power. The collected data offer valuable insights into how such tests affect military personnel in the subsequent days, particularly in key tasks such as jumping, overcoming obstacles, moving with agility and coordination, carrying and dragging heavy loads, and running long distances. Despite the observed enhancements in muscular strength and power, caution should be exercised when performing submaximal or maximal activities following such tests, due to the significant neuromuscular fatigue and potential deterioration in motor control [4], which could ultimately increase the risk of musculoskeletal injury.

## Figures and Tables

**Table 1:** Results of performance variables at different time points.

	PRE	POST	24 H POST	48 H POST	ES^1^	ES^2^	ES^3^
ROF [points]	2.34 ± 0.81	8.95 ± 0.76 ‡	4.12 ± 1.02 ‡Ψ	3.59 ± 0.63 ‡ΨŦ	−7.18	−1.39	−1.17
BM [kg]	78.43 ± 7.99	78.00 ± 7.94 ‡	77.97 ± 8.06 ‡	78.44 ± 8.11 ¥Ͳ	0.94	0.78	−0.02
BL [mmol/L]	2.20 ± 0.00 [17]	10.53 ± 3.91 ‡	ND	ND	−2.13	ND	ND
PUmax [reps]	17.28 ± 4.73	15.34 ± 4.41 ‡	15.00 ± 3.66 ‡	15.66 ± 3.67 ‡Ᵹ	0.75	0.79	0.74
DHGS [kg]	52.04 ± 7.60	50.60 ± 8.04	51.17 ± 7.17	51.26 ± 6.64	0.30	0.23	0.18
NDHGS [kg]	49.12 ± 6.57	48.76 ± 7.43	48.86 ± 6.64	49.46 ± 6.78	0.08	0.07	−0.08
IMTP [N]	2590.07 ± 301.98	2633.31 ± 340.13	2427.10 ± 301.71 ‡Ψ	2471.79 ± 311.95 †Ψ	−0.25	1.04	0.61
VJ CMJ [cm]	31.35 ± 5.00	32.12 ± 5.45	30.33 ± 5.12 *¥	30.25 ± 4.97 *¥	−0.29	0.40	0.41
PP CMJ [W]	3690.31 ± 691.40	3560.24 ± 660.33	3410.76 ± 486.57	3501.48 ± 521.17	0.16	0.46	0.28
RFDEcc CMJ [N/s]	3592.31 ± 1743.92	3055.97 ± 1833.50	3896.31 ± 1759.16 §	4295.83 ± 1533.59 *Ψ	0.36	−0.20	−0.50
RFDCon CMJ [N/s]	1436.45 ± 855.45	1568.76 ± 1168.78	1167.28 ± 813.54	1140.21 ± 1046.26	−0.11	0.32	0.25
PEcc CMJ [N]	1628.83 ± 319.79	1503.79 ± 326.18 *	1632.24 ± 256.82 §	1686.00 ± 249.49 Ψ	0.39	−0.02	−0.36
PCon CMJ [N]	1743.07 ± 228.25	1734.14 ± 233.22	1729.48 ± 214.97	1747.31 ± 204.42	0.07	0.13	−0.04

BM: body mass; CMJ: countermovement jump; DHGS: dominant hand grip strength; ES^1^: effect size pre–post; ES^2^: effect size pre–24 h post; ES^3^: effect size pre–48 h post; IMTP: isometric mid-thigh pull; ND: not determined; NDHGS: non-dominant hand grip strength; PCon: peak concentric force; PEcc: peak eccentric force; PP: peak power; RFD: rate of force development; ROF: rating of fatigue; and VJ: maximum vertical jump height from take-off. Values are presented as means ± SD. The following symbols indicate significant differences compared to values: (a) pre: (*): significant difference (*p* ≤ 0.05); (†): very significant difference (*p* ≤ 0.01); (‡): extremely significant difference (*p* ≤ 0.001); (b) post: (§): significant difference (*p* ≤ 0.05); (¥): very significant difference (*p* ≤ 0.01); (Ψ): extremely significant difference (*p* ≤ 0.001); and (c) post 24 h: (Ᵹ): significant difference (*p* ≤ 0.05); (Ŧ): very significant difference (*p* ≤ 0.01); (Ͳ): extremely significant difference (*p* ≤ 0.001).

**Table 2:** Report on incidents and perceived effort following the 30 km endurance march.

	T1 (0–1 km)	T2 (1–2 km)	T3 (2–3 km)	T4 (3–4 km)	T5 (4–5 km)	ES^1^	ES^2^	ES^3^	ES^4^
Fatigue	14.48 ± 15.26	23.79 ± 25.83 †	40.34 ± 26.92 ‡Ψ	67.24 ± 28.52 ‡ΨͲ	75.52 ± 28.48 ‡ΨͲƱ	−0.57	−1.35	−2.12	−2.25
Muscle pain	13.45 ± 15.18	16.55 ± 20.58	25.86 ± 29.70 ‡Ψ	37.24 ± 29.63 ‡ΨŦ	45.52 ± 37.38 ‡ΨͲ	−0.33	−0.66	−1.09	−1.11
Joint pain	11.38 ± 14.81	13.45 ± 18.95	18.62 ± 28.25 *§	25.86 ± 33.76 †¥Ᵹ	27.93 ± 38.39 †¥Ᵹ	−0.27	−0.42	−0.58	−0.58
Shortness of breath	12.41 ± 15.04	22.76 ± 27.37 †	34.14 ± 32.79 ‡Ψ	42.41 ± 37.19 ‡ΨꝽ	60.00 ± 34.95 ‡ΨͲΣ	−0.56	−0.96	−1.13	−1.75
Excessive sweating	13.45 ± 17.17	25.86 ± 23.68 ‡	37.24 ± 27.37 ‡¥	61.03 ± 28.33 ‡ΨͲ	73.45 ± 23.49 ‡ΨͲƱ	−0.83	−1.09	−1.75	−2.26
Muscle tremor	11.38 ± 14.81	14.48 ± 20.63	17.59 ± 27.21	22.76 ± 31.72 †§	23.79 ± 32.45 †¥Ᵹ	−0.33	−0.37	−0.52	−0.57
Palpitations	10.34 ± 14.51	15.52 ± 24.87	26.90 ± 33.39 ‡¥	30.00 ± 36.74 ‡Ψ	28.97 ± 37.16 ‡¥	−0.37	−0.67	−0.81	−0.76

ES^1^: effect size T1–T2; ES^2^: effect size T1–T3; ES^3^: effect size T1–T4; ES^4^: effect size T1–T5. Values are presented as means ± SD. The following symbols indicate significant differences compared to values: (a) 0–1 km: (*): significant difference (*p* ≤ 0.05); (†): very significant difference (*p* ≤ 0.01); (‡): extremely significant difference (*p* ≤ 0.001); (b) 1–2 km: (§): significant difference (*p* ≤ 0.05); (¥): very significant difference (*p* ≤ 0.01); (Ψ): extremely significant difference (*p* ≤ 0.001); (c) 2–3 km: (Ᵹ): significant difference (*p* ≤ 0.05); (Ŧ): very significant difference (*p* ≤ 0.01); (Ͳ): extremely significant difference (*p* ≤ 0.001); and (d) 3–4 km: (Ʊ): very significant difference (*p* ≤ 0.01); (Σ): extremely significant difference (*p* ≤ 0.001).

## Data Availability

Data supporting the study’s findings are available from the corresponding author upon reasonable request, but are not publicly shared due to ethical and privacy considerations.
